# Arsenic trioxide-induced cardiotoxicity: insights into the oxidative stress-inflammation positive feedback loop, translational biomarkers and targeted cardioprotective strategies— a narrative review

**DOI:** 10.3389/fonc.2026.1954263

**Published:** 2026-10-12

**Authors:** Haiyan Wu, Jian Lv, Xiaoyang Hu

**Affiliations:** 1 Graduate School, Heilongjiang University of Chinese Medicine, Harbin, Heilongjiang, China; 2 School of Basic Medical Science, Heilongjiang University of Chinese Medicine, Harbin, Heilongjiang, China

**Keywords:** arsenic trioxide, drug-induced cardiotoxicity, NF-κB signaling pathway, NLRP3 inflammasome, oxidative stress-inflammation positive feedback loop, translational biomarkers

## Abstract

Arsenic trioxide (ATO) is a first-line chemotherapeutic agent that achieves a complete remission rate above 90% in newly diagnosed acute promyelocytic leukemia, with indications expanding to multiple hematological malignancies and solid tumors. However, dose-dependent cardiotoxicity—manifesting as acute QTc prolongation and malignant arrhythmia, as well as chronic myocardial hypertrophy, interstitial fibrosis and progressive heart failure—remains a critical clinical barrier that limits its therapeutic window, and no approved targeted cardioprotective regimens are currently available in clinical practice. This narrative Mini Review proposes an integrated mechanistic framework centered on the oxidative stress–inflammation positive feedback loop, which complements the conventional view of two independent pathogenic pathways. In this framework, ATO-induced mitochondrial dysfunction and impaired Nrf2 antioxidant defense initiate oxidative stress as the upstream trigger; ROS subsequently drives inflammatory activation via NF-κB and NLRP3 inflammasome pathways, while sustained inflammation in turn aggravates oxidative damage by suppressing Nrf2 activity and disrupting mitochondrial homeostasis. This proposed self-amplifying loop exhibits spatiotemporal heterogeneity. It appears incompletely established in acute electrophysiological injury, but may serve as a potential mechanistic contributor to chronic structural remodeling. Based on this framework, this work summarizes dual-target cardioprotective strategies that preserve ATO’s anti-tumor efficacy, and evaluates translational biomarkers for early risk stratification. Collectively, this review provides a mechanistic foundation for precision cardioprotection and clinical safety optimization of ATO-based chemotherapy.

## 1 Introduction

Arsenic trioxide (ATO) is an arsenic-containing therapeutic agent with a long application history in traditional medicine. Accumulating modern medical evidence has validated its outstanding efficacy in the treatment of acute promyelocytic leukemia (APL). It can achieve a complete remission rate of more than 90% in newly diagnosed APL patients and substantially improve their long-term survival outcomes (1, 2). As the understanding of its anti-tumor mechanisms continues to deepen, the clinical indications of ATO have gradually expanded to multiple myeloma, myelodysplastic syndromes, and solid tumors represented by neuroblastoma. ATO has therefore become a broad-spectrum anti-tumor agent exerting effects of differentiation induction, apoptosis promotion and anti-angiogenesis (3, 4). In multicenter clinical studies on pediatric high-risk neuroblastoma, the combination of ATO and chemotherapy increases the objective response rate after the completion of induction therapy to 86.36%. This therapeutic regimen presents a generally favorable tolerability profile and holds promising clinical application potential (4).

With the expanding clinical application of ATO, cardiotoxicity has gradually emerged as a critical factor limiting its dosing and treatment duration. ATO-induced cardiac adverse events exhibit heterogeneous clinical manifestations and can be classified into acute and chronic injury patterns. Acute cardiotoxicity typically occurs within days to weeks after drug administration, with cardiac electrophysiological abnormalities as the core manifestation. These abnormalities include QT interval (QTc) prolongation, sinus tachycardia and atrioventricular block, and severe cases may develop torsades de pointes (TdP) or even sudden cardiac death (5–7). Clinical data indicate that at a daily dose of 0.15 mg/kg, 2 of 15 patients experienced dose-limiting QTc prolongation, pneumonia, or neuropathic pain. At 0.2 mg/kg daily, 2 of 4 patients developed dose-limiting QTc prolongation or pancreatitis (8). Long-term exposure to arsenic can also lead to cardiovascular disease. It is characterized by cardiomyocyte hypertrophy, abnormal cardiac contractions and ventricular remodeling, and eventually progresses to systolic dysfunction and congestive heart failure (2, 9). In addition, concomitant administration of ATO with other cardiotoxic anti-neoplastic agents including anthracyclines and tyrosine kinase inhibitors exerts additive cardiac injury effects and significantly elevates the incidence of cardiovascular adverse events in patients (10–12).

Previous investigations into the mechanisms underlying ATO-induced cardiotoxicity have largely centered on single signaling pathways. The oxidative stress theory is the most widely recognized mechanistic framework. ATO causes oxidative injury to cardiomyocytes by promoting reactive oxygen species (ROS) generation and depleting endogenous antioxidant defenses (1, 13). Meanwhile, accumulating evidence demonstrates that inflammatory responses play a critical role in ATO-induced myocardial damage. The release of pro-inflammatory cytokines and infiltration of innate immune cells jointly contribute to the impairment of myocardial structure and function (14, 15). However, most existing studies regard oxidative stress and inflammation as two parallel independent pathogenic processes. Limited attention has been paid to their interactive regulation and cascade amplification effects during cardiotoxicity progression. In fact, oxidative stress functions as an upstream signal to trigger inflammasome assembly and activation of pro-inflammatory pathways. In turn, inflammatory responses further aggravate oxidative damage by upregulating oxidase expression. The positive feedback loop formed by the two processes is indicative of a putative regulatory axis that may contribute to the sustained progression of cardiotoxicity, though its definitive causal role remains to be established by targeted interventional studies. In addition, current studies provide insufficient elucidation of the mechanistic heterogeneity between acute electrophysiological injury and chronic structural remodeling. This knowledge gap impedes the development of stratified clinical intervention strategies.

Guided by unmet clinical needs, the present review comprehensively integrates available experimental evidence and constructs a proposed integrative mechanistic framework of ATO-induced cardiotoxicity, with the oxidative stress-inflammation positive feedback loop as a putative regulatory axis. This review first defines the dual phenotypes and pathological features of ATO-induced cardiotoxicity, and then hierarchically elucidates the initiating mechanism of oxidative stress and the amplification effect of inflammatory responses. Particular focus is placed on deciphering the molecular basis of their interactive regulation and its spatiotemporal differences between acute and chronic cardiac injuries. On this basis, this review critically summarizes cardioprotective strategies targeting the oxidative stress-inflammation axis and comparatively analyzes the functional characteristics and translational value of distinct intervention regimens. Finally, it identifies technical bottlenecks in current research and proposes prospective research directions, which is intended to provide a theoretical framework for developing highly effective and precise protective strategies against ATO-induced cardiotoxicity.

Evidence-level notation used throughout this review. To help readers judge the strength of support for each mechanistic claim, we adopt a three-tier evidence classification: (i) [Direct cardiac evidence] — findings obtained from ATO-exposed cardiomyocytes, cardiac tissue, or *in vivo* ATO cardiotoxicity models; (ii) [Indirect cardiac evidence] — findings from ATO-exposed cardiac tissues showing downstream readouts (e.g., cytokine levels, caspase activity) that are consistent with a pathway but do not directly validate the pathway itself; and (iii) [Extrapolated evidence] — findings from non-cardiac ATO models (e.g., leukemia cells, macrophages, neuronal cells) or from other cardiovascular disease models, where the mechanism is biologically plausible but direct ATO-cardiac validation is lacking. These labels are applied in the text below and are visually encoded in **Figure 1** (solid vs. dashed lines; filled vs. outlined shapes).

**Figure 1:**
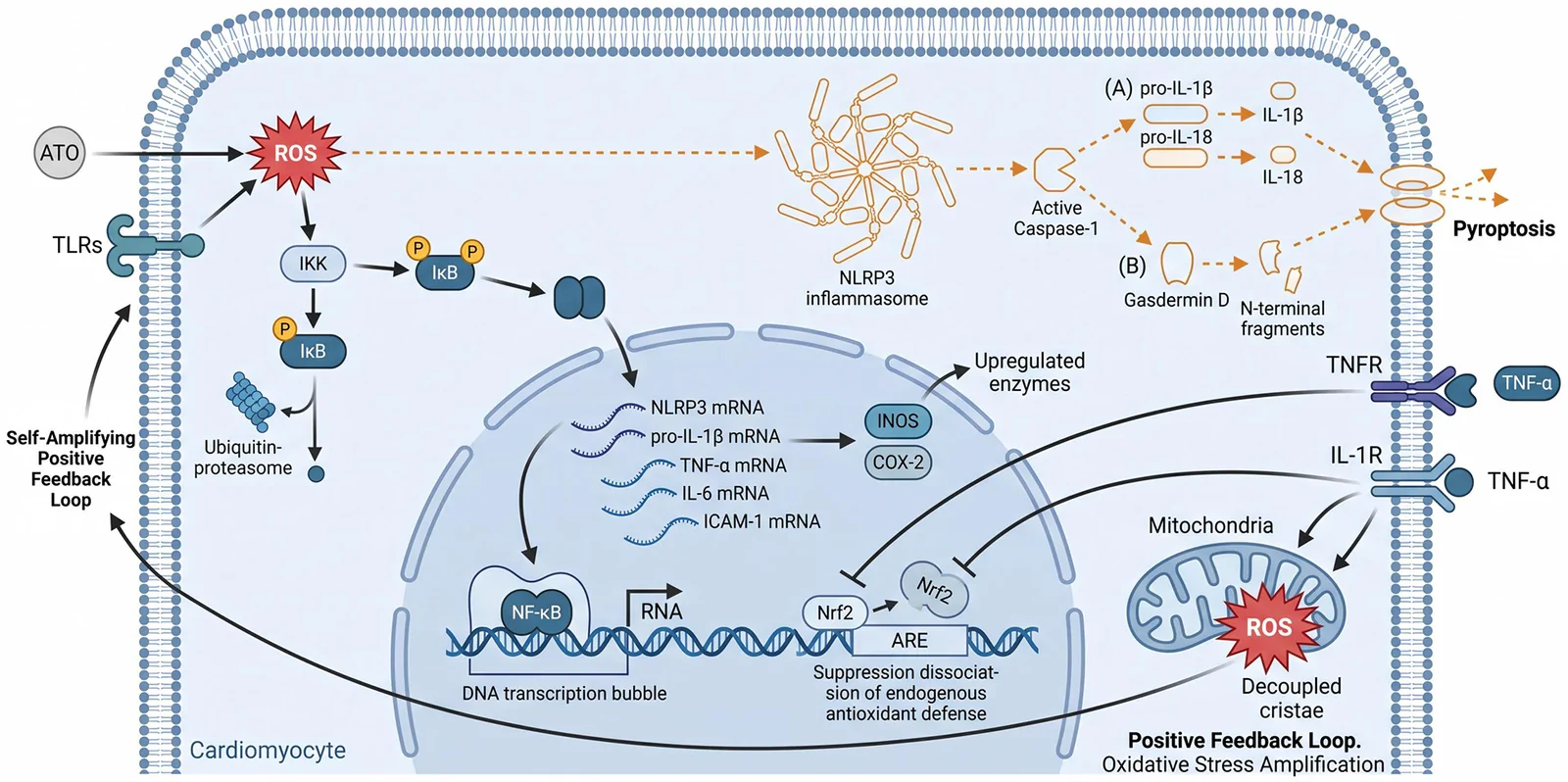
Inflammation as the amplification step and positive feedback loop in ATO-induced cardiotoxicity. Arsenic trioxide (ATO) induces reactive oxygen species (ROS) generation. ROS and Toll-like receptors (TLRs) activate IKK, which phosphorylates IκB, leading to its ubiquitin-proteasomal degradation and NF-κB release. NF-κB translocates to the nucleus to transcribe NLRP3, pro-IL-1β, TNF-α, IL-6, and ICAM-1 mRNAs, upregulating cytosolic iNOS and COX-2. Upstream ROS triggers NLRP3 inflammasome assembly and Caspase-1 activation. Active Caspase-1 cleaves pro-IL-1β/18 into mature cytokines **(A)** and Gasdermin D into N-terminal fragments **(B)**. These fragments form membrane pores, inducing pyroptosis and the extracellular release of cytokines and DAMPs. Secreted cytokines bind to membrane receptors (TNFR/IL-1R), sending a suppressive signal that dissociates Nrf2 from the ARE, impairing endogenous antioxidant defenses. Simultaneously, inflammatory signaling causes mitochondrial cristae decoupling and a massive secondary ROS burst. This new ROS re-triggers the priming phase, completing a self-amplifying loop. Solid black lines & filled shapes: Indicate mechanisms with direct experimental evidence in ATO-exposed cardiac tissue (e.g., NF-κB activation, Nrf2 suppression). Solid lines with blunt T-bars represent inhibitory interactions. Dashed orange lines & outlined (hollow) shapes: Indicate proposed or extrapolated mechanisms derived primarily from non-cardiac models, where direct cardiac validation remains limited (e.g., inflammasome assembly, pyroptosis).

## 2 Clinical phenotypes and pathological features of ATO-induced cardiotoxicity

ATO-induced cardiotoxicity exhibits time-dependent phenotypic heterogeneity. We propose a working model in which two injury subtypes are underpinned by different core molecular mechanisms: acute electrophysiological dysfunction and chronic structural remodeling. This phase distinction is based on cross-sectional preclinical and clinical observations, and direct longitudinal evidence tracking the same cardiac compartment from acute to chronic phases remains limited.

### 2.1 Acute electrophysiological cardiotoxicity: ion channel dysfunction as the core mechanism

Acute cardiotoxicity generally develops within hours to weeks after drug administration. It is characterized by core abnormalities in myocardial electrical activity, and its pathogenesis is closely associated with the rapid regulatory effects of ATO on myocardial ion channels (16). Multiple clinical studies have confirmed that intravenous infusion of ATO leads to dose-dependent prolongation of the corrected QTc (5, 7). The risk of malignant arrhythmia is significantly elevated when QTc exceeds 500 ms or prolongs by more than 60 ms relative to baseline levels (17, 18). In addition to repolarization abnormalities, ATO can also induce sinus tachycardia and varying degrees of conduction block (19). Reversible elevation of myocardial enzymes has been reported in a subset of patients (4). Notably, acute cardiotoxicity is mostly dose-dependent and reversible, and can gradually resolve after drug withdrawal or symptomatic management. Nevertheless, severe TdP may still trigger sudden cardiac death, which represents the most concerning safety issue in clinical pharmacotherapy (20, 21).

### 2.2 Chronic structural cardiotoxicity: mitochondrial dysfunction, cell death and interstitial fibrosis

Chronic cardiotoxicity typically develops after long-term maintenance therapy or multiple treatment cycles, and is primarily characterized by irreversible structural remodeling of the myocardium. Animal experimental evidence indicates that 4 to 6 weeks of continuous ATO administration in rats leads to elevated cardiac weight index, ventricular wall thickening and pathological manifestations of myocardial hypertrophy (14, 22). With prolonged ATO exposure, progressive interstitial fibrosis and progressive cardiomyocyte loss occur in the myocardium. These pathological changes eventually result in impaired ventricular diastolic and systolic function, and may even progress to heart failure (23). Clinical follow-up data demonstrate that the incidence of subclinical cardiac dysfunction is significantly higher in patients receiving long-term ATO treatment than in the general population. This cardiac damage may persist even after drug discontinuation, and acts as a critical contributor to long-term cardiovascular events among cancer survivors (24).

With respect to myocardial parenchymal cells, ATO is established to induce apoptosis, and may also engage an inflammation-associated cell-death program consistent with pyroptosis. Apoptosis represents the primary injury pattern in the early stage, supported by cardiomyocyte nuclear pyknosis, chromatin margination, apoptotic body formation, caspase-3/caspase-12 activation and elevated Bax/Bcl-2 ratio (25). By contrast, evidence for canonical pyroptosis in ATO-exposed cardiomyocytes is indirect. Canonical pyroptosis requires caspase-1-dependent cleavage of gasdermin D, formation of N-terminal membrane pores, and subsequent membrane rupture; while elevated mature IL-1β/IL-18 and increased caspase-1 activity have been observed in ATO-exposed cardiac tissue, direct demonstration of gasdermin D cleavage, pore formation, or pyroptotic membrane rupture in ATO cardiac models has not been reported. The reference (26) cited here describes ROS-dependent pyroptosis in a non-cardiac hepatic cell line (NCTC-1469), and is therefore relevant as supportive rather than cardiac-specific evidence.

At the myocardial interstitial level, ATO induces activation and proliferation of cardiac fibroblasts and promotes collagen synthesis and deposition. These pathological alterations drive the progression of myocardial interstitial fibrosis and perivascular fibrosis. Masson’s trichrome staining demonstrates that the interstitial collagen volume fraction in the myocardium of ATO-treated rats is significantly elevated. The severity of fibrosis presents a positive correlation with the administered dose of ATO (14). In addition, ATO can damage vascular endothelial cells and impair endothelial barrier function. It also promotes adhesion molecule expression and leukocyte adhesion, which further exacerbates inflammatory infiltration and ischemic injury in myocardial tissues (27).

### 2.3 Proposed reciprocal promotion between acute electrical remodeling and chronic structural remodeling

ATO-induced cardiac dysfunction is thought to arise from the combined effects of electrical remodeling and structural remodeling, which are proposed to be underpinned by distinct molecular mechanisms. Electrical remodeling serves as the core pathological basis of acute cardiotoxicity, and it is primarily attributed to aberrant expression and dysfunction of myocardial ion channels. Accumulating studies have confirmed that ATO inhibits the protein trafficking and membrane expression of the rapid delayed rectifier potassium (IKr) channel encoded by the human ether-a-go-go related gene (hERG). This suppression reduces IKr current density and prolongs myocardial action potential duration, which represents the predominant ionic mechanism underlying QTc prolongation (28–30). In addition, ATO downregulates the expression of the inward rectifier potassium channel Kir2.1 and decreases IK1 current, which further exacerbates repolarization abnormalities (31). Beyond potassium channels, ATO disrupts intracellular calcium homeostasis and triggers intracellular calcium overload in cardiomyocytes. This disturbance impairs myocardial excitation-contraction coupling and also initiates afterdepolarization as well as arrhythmia (32, 33).

Structural remodeling constitutes the pathological basis of chronic cardiac dysfunction. Cardiomyocyte loss, myocardial interstitial fibrosis and abnormal remodeling of the extracellular matrix collectively give rise to increased ventricular wall stiffness and decreased myocardial contractility. Meanwhile, long-term oxidative stress and inflammatory stimulation damage the structure and function of myocardial mitochondria. This results in insufficient ATP production and further impairs myocardial systolic and diastolic function (34, 35). Electrical remodeling and structural remodeling are not entirely independent processes. Persistent electrophysiological abnormalities can induce cardiomyocyte calcium overload and metabolic disorders, which accelerate structural injury. In turn, structural remodeling alters the homogeneity of myocardial electrical conduction and increases susceptibility to arrhythmia. The two processes exert mutually reinforcing effects and jointly drive the progression of cardiotoxicity (6).

## 3 Oxidative stress as the initiating step of ATO-induced cardiotoxicity

Oxidative stress refers to a state of imbalance between ROS production and the endogenous antioxidant defense system. Excessive ROS inflicts damage to cellular structure and function via oxidative modification of proteins, lipids and nucleic acids. In the setting of ATO-induced cardiotoxicity, oxidative stress represents one of the earliest detectable cellular events. It acts both as a direct manifestation of ATO cytotoxicity and as an upstream trigger for subsequent inflammatory responses and cell death, thereby exerting an initiating effect throughout the entire toxicity cascade (**Figure 2**).

**Figure 2:**
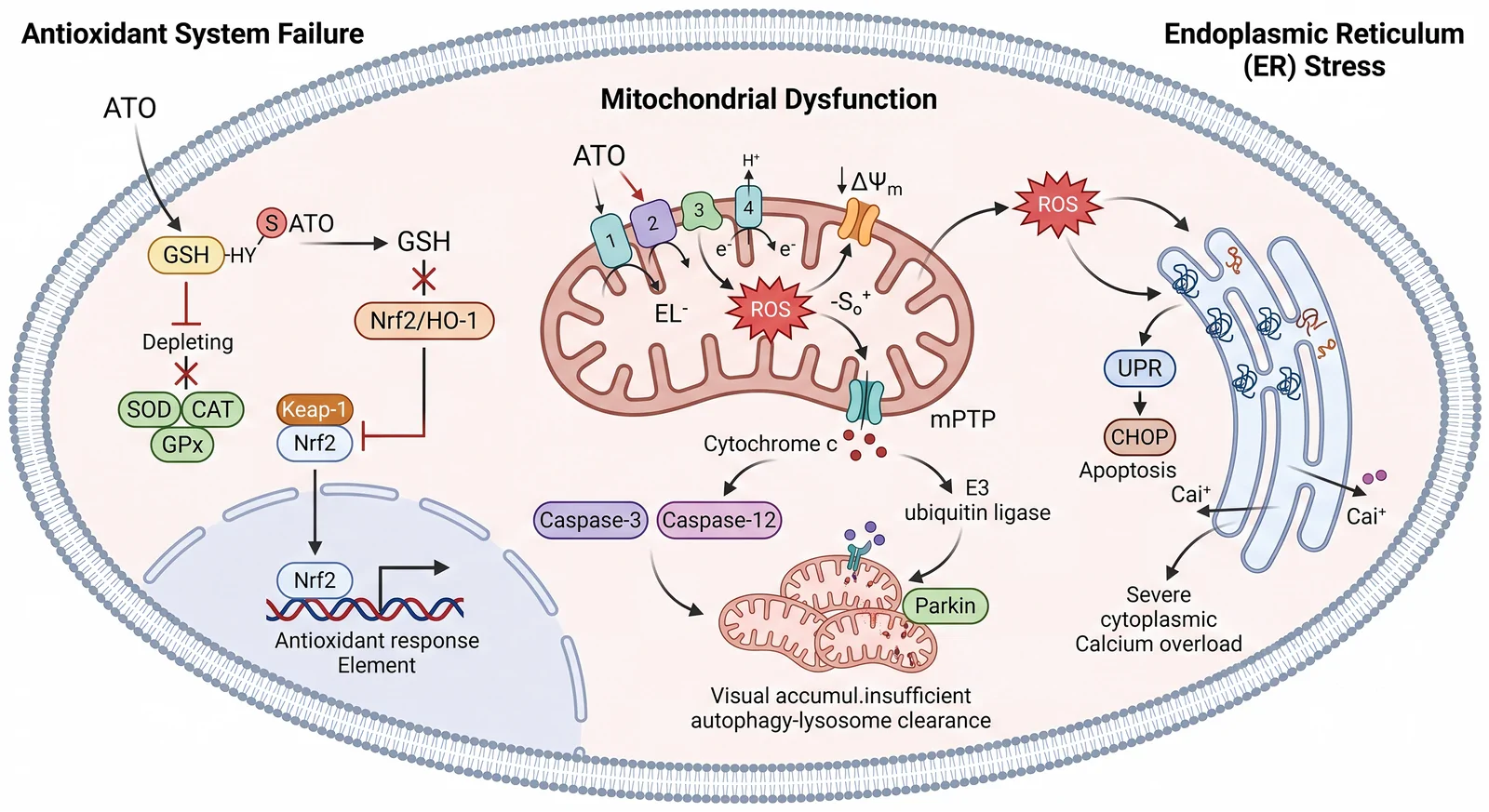
The role of oxidative stress in ATO-induced cardiotoxicity. Arsenic Trioxide (ATO) is shown entering the cell membrane and directly causing several parallel but interconnected failure cascades. First, it initiates Antioxidant System Failure by binding directly to and depleting reduced glutathione (GSH), while simultaneously suppressing enzymatic antioxidants like superoxide dismutase (SOD), catalase (CAT), and glutathione peroxidase (GPx), leaving the cell vulnerable. This state is further compounded by the blockade of the Nrf2/HO-1 signaling pathway, where Keap-1 is explicitly shown sequestering Nrf2 in the cytoplasm, preventing its nuclear translocation. The core mechanism is Mitochondrial Dysfunction, where ATO targets respiratory chain complexes on the inner membrane, leading to severe electron leakage and massive reactive oxygen species (ROS) generation, specifically superoxide anions. This process causes a dramatic drop in mitochondrial membrane potential (ΔΨm), opens the mitochondrial permeability transition pore (mPTP), and releases cytochrome c into the cytoplasm, triggering intrinsic apoptosis. Meanwhile, damaged mitochondria accumulate due to insufficient autophagy clearance, despite recruitment of the E3 ubiquitin ligase Parkin. Simultaneously, increased ROS attack the nearby Endoplasmic Reticulum (ER), causing Endoplasmic Reticulum Stress marked by the accumulation of misfolded proteins. This triggers the unfolded protein response (UPR) and CHOP-mediated apoptosis, along with a leakage of calcium ions (Ca²^+^) into the cytoplasm, resulting in severe cytoplasmic calcium overload that further exacerbates mitochondrial stress. Ultimately, these parallel signals converge into a unified intrinsic apoptotic pathway. The mitochondrial dysfunction, Nrf2/Keap1 antioxidant suppression, and GSH depletion cascades are supported by direct experimental evidence in ATO-exposed cardiomyocytes and myocardial tissue. The endoplasmic reticulum stress (ERS)-mediated CHOP/caspase-12 apoptosis branch is partially extrapolated from ATO-treated non-cardiac cell models (leukemia and lung cancer cells) and requires further cardiac-specific validation. Black Arrow (→): Increased; T Arrow (T):Inhibited.

### 3.1 Mitochondrial dysfunction

Mitochondria serve as the core site of energy metabolism in cardiomyocytes, and also act as the primary source of ATO-induced ROS generation. ATO impairs mitochondrial structure and function through multiple molecular targets, thereby triggering the oxidative stress cascade. First, ATO suppresses the activity of mitochondrial respiratory chain complexes and interferes with electron transport, leading to electron leakage and superoxide anion generation. This process constitutes a major source of ROS production in cardiomyocytes. Direct evidence for specific complex suppression has been obtained in H9c2 cardiomyoblasts (36); findings from non-cardiac leukemia models are consistent with this mechanism but require cardiac-specific confirmation. Accumulating studies have confirmed that ATO treatment markedly elevates mitochondrial superoxide levels in H9c2 cardiomyocytes. This alteration is accompanied by a reduced respiratory control ratio and impaired ATP synthesis (25, 37). Second, ATO decreases mitochondrial membrane potential (ΔΨm) and induces opening of the mitochondrial permeability transition pore (mPTP). This has been directly demonstrated in isolated mitochondria exposed to arsenic (38); confirmation in intact cardiomyocytes and *in vivo* myocardial tissue remains more limited. mPTP opening leads to mitochondrial matrix swelling, outer membrane rupture, and release of pro-apoptotic factors such as cytochrome c into the cytoplasm, which may activate the intrinsic apoptotic pathway.

In addition, mitophagy serves as a vital cellular mechanism for eliminating damaged mitochondria and maintaining mitochondrial homeostasis. ATO can disrupt this homeostatic process and aggravate mitochondrial dysfunction. Accumulating studies have revealed that ATO treatment activates the ubiquitin-proteasome system and facilitates the recruitment of the E3 ubiquitin ligase Parkin to mitochondria. This event initiates the degradation process through ubiquitination of mitochondrial substrates. However, activation of the autophagy-lysosome pathway is relatively insufficient under ATO exposure. This defect prevents the effective clearance of damaged mitochondria and results in the accumulation of dysfunctional mitochondria (5). This imbalance in mitochondrial quality control further amplifies the magnitude of oxidative stress. It eventually forms a vicious cycle where mitochondrial damage promotes ROS production, and the accumulated ROS in turn triggers more severe mitochondrial injury.

### 3.2 Imbalance of the endogenous antioxidant defense system

Cardiomyocytes are equipped with a well-established antioxidant defense system composed of enzymatic and non-enzymatic antioxidants, which work in concert to maintain intracellular redox homeostasis. ATO disrupts this homeostatic state and exacerbates oxidative damage by depleting antioxidant substrates and suppressing the activity of antioxidant enzymes. Reduced glutathione (GSH) is the most predominant non-enzymatic antioxidant within cells. It directly scavenges ROS and serves as a coenzyme for glutathione peroxidase (GPx). ATO binds to the sulfhydryl groups of GSH and induces GSH depletion. Meanwhile, it reduces the activity of glutathione reductase and blocks GSH regeneration, ultimately resulting in the disturbance of intracellular redox status (1, 39). Multiple *in vivo* animal studies have verified that GSH content and the GSH/GSSG ratio in myocardial tissue are significantly reduced following ATO administration. The magnitude of this reduction exhibits a positive correlation with the severity of myocardial injury (1, 13).

Within the enzymatic antioxidant system, superoxide dismutase (SOD), catalase (CAT) and GPx serve as core components. They are responsible for scavenging superoxide anions, hydrogen peroxide and lipid peroxides, respectively. ATO markedly reduces the activity of these antioxidant enzymes through oxidative modification of active sites on enzyme proteins and downregulation of enzyme protein expression. This functional impairment leads to the accumulation of uncleared ROS (1, 40). Nuclear factor erythroid 2-related factor 2 (Nrf2) serves as the master transcription factor governing cellular endogenous antioxidant responses. Under homeostatic physiological conditions, Kelch-like ECH-associated protein 1 (Keap1) functions as a key cytoplasmic negative regulator: Keap1 binds to Nrf2 and mediates its continuous ubiquitination and proteasomal degradation to keep Nrf2 at low basal abundance. Upon oxidative stress insult, reactive-oxygen-species-driven cysteine modification of Keap1 disrupts the Keap1-Nrf2 protein complex. Stabilized Nrf2 subsequently translocates into the nucleus, heterodimerizes with small Maf proteins, and binds to antioxidant-response-element (ARE) sequences on promoter regions. This transcriptional program activates the expression of downstream antioxidant and phase-II detoxifying effector genes, among which heme oxygenase-1 (HO-1) and NAD(P)H quinone dehydrogenase 1 (NQO1) are well-characterized representatives. Studies have revealed that ATO suppresses Nrf2 nuclear translocation and transcriptional activity. Consequently, ARE-driven downstream antioxidant effectors HO-1 and NQO1 are markedly downregulated, which impairs the intrinsic antioxidant defense capacity of cardiomyocytes. It should be emphasized that Keap1 is not an Nrf2-controlled downstream effector; instead, it constitutes the primary upstream negative modulator of this cascade. Notably, cardiomyocytes inherently exhibit limited Nrf2-inducible activity, which partially explains the high vulnerability of myocardial tissue toward ATO-elicited oxidative injury (41).

### 3.3 Endoplasmic reticulum stress-mediated oxidative damage

The endoplasmic reticulum is a critical organelle that mediates protein folding and intracellular calcium storage. A close reciprocal regulatory relationship exists between oxidative stress and endoplasmic reticulum stress (ERS). ATO induces ERS via multiple mechanisms. In turn, activated ERS further amplifies oxidative stress, forming an additional localized vicious cycle.

On the one hand, ATO-induced ROS accumulation causes oxidative modification of folding enzymes and molecular chaperones within the endoplasmic reticulum. This alteration drives the accumulation of unfolded and misfolded proteins in the endoplasmic reticulum lumen, which subsequently triggers the unfolded protein response (UPR) (42). On the other hand, ATO disrupts endoplasmic reticulum calcium homeostasis. It increases calcium release from the sarcoplasmic reticulum and reduces calcium reuptake, leading to cytoplasmic calcium overload. Meanwhile, decreased calcium concentration in the endoplasmic reticulum lumen impairs the function of calcium-dependent molecular chaperones and exacerbates protein folding defects (42, 43).

The UPR activated in the initial phase of ERS serves as a cytoprotective cellular response. It restores endoplasmic reticulum homeostasis by inhibiting protein synthesis and upregulating the expression of molecular chaperones. However, persistent or severe ERS may drive a shift toward pro-apoptotic signaling. It activates apoptotic pathways involving CHOP and caspase-12, which may ultimately contribute to cardiomyocyte death. It should be noted that direct evidence for CHOP/caspase-12-mediated apoptosis in ATO-exposed cardiomyocytes remains limited; the cited studies were conducted in ATO-treated leukemia cells (44) and A549 lung cancer cells (45), and these findings are extrapolated to cardiac tissue. Cardiac-specific validation of this ERS-apoptosis branch is warranted. Meanwhile, ERS promotes ROS generation through multiple mechanisms, such as activating NADPH oxidase and disrupting mitochondrial function, which further exacerbates cellular oxidative damage. Accumulating studies have confirmed that cardioprotective agents represented by salvianolic acid B significantly attenuate ATO-induced myocardial injury by improving endoplasmic reticulum calcium homeostasis and alleviating ERS severity. These findings inversely verify the critical involvement of ERS in the pathogenesis of ATO-induced cardiotoxicity (2).

## 4 Inflammation as the amplification step of ATO-induced cardiotoxicity

Inflammatory responses serve as defensive reactions of the organism against tissue injury and infection. Excessive and sustained inflammation, however, can inflict secondary damage to tissues. In the setting of ATO-induced cardiotoxicity, inflammation does not function as an independent pathogenic factor. It is activated downstream of oxidative stress, and further amplifies myocardial injury by releasing abundant pro-inflammatory mediators and recruiting immune cells. In the chronic phase of ATO-induced cardiotoxicity, sustained inflammation may act as a critical amplification step that contributes to the progression from reversible functional injury toward chronic structural damage. In contrast, acute electrophysiological injury is dominated by direct ion channel dysfunction, and the full oxidative stress–inflammation feedback loop appears incompletely established at this early stage (**Figure 1**).

### 4.1 Inflammasome activation and pyroptosis

Inflammasomes are multiprotein complexes located in the cytoplasm and serve as critical molecular platforms linking innate immunity and inflammatory responses. Among all identified inflammasome subtypes, the NLRP3 inflammasome is the most extensively investigated and exhibits the strongest association with oxidative stress.

Activation of the NLRP3 inflammasome follows a two-step process composed of a priming phase and an activation phase. The priming phase is mediated by pattern recognition receptors such as Toll-like receptors (TLRs). It upregulates the expression of key inflammasome components including NLRP3 and pro-IL-1β through the NF-κB signaling pathway. The activation phase is triggered by diverse stimuli such as ATP, ROS and crystalline substances. These stimuli induce NLRP3 oligomerization and promote recruitment of the adaptor protein ASC and the effector protein pro-caspase-1, ultimately leading to the assembly of functional inflammasomes (46).

It should be noted that direct experimental evidence specifically validating NLRP3 inflammasome activation and gasdermin D-mediated pyroptosis in ATO-induced myocardial injury remains sparse. Current supportive evidence for this pathway is derived from three levels with different strengths of validation.

[Indirect cardiac evidence] First, ATO-exposed cardiac tissues show elevated mature IL-1β and IL-18, accompanied by increased caspase-1 activity (33, 43). These readouts are consistent with inflammasome activation but, importantly, do not by themselves establish canonical pyroptosis: direct demonstration of gasdermin D cleavage, N-terminal pore formation, or pyroptotic membrane rupture in ATO cardiomyocytes or myocardium has not yet been reported. These findings therefore support inflammasome involvement but leave the downstream cell-death mode — pyroptotic versus non-pyroptotic caspase-1 signaling — unresolved. [Extrapolated evidence — non-cardiac ATO models] Second, findings from non-cardiac ATO toxicity models offer further indirect evidence. In RAW 264.7 macrophages exposed to ATO, glucogallin was shown to attenuate inflammatory injury via inhibiting the NF-κB/NLRP3 signaling axis, confirming that ATO can trigger NLRP3 inflammasome activation in immune cells (47). Studies on ATO-induced neurotoxicity also demonstrated that inflammasome activation acts as a conserved downstream event of oxidative stress in ATO-related multi-organ damage. [Extrapolated evidence — other cardiovascular models] Third, evidence from other cardiovascular disease models consistently shows that ROS-driven NLRP3 inflammasome assembly and pyroptosis represent a general pathogenic mechanism of myocardial injury, and selective NLRP3 inhibitors exert robust cardioprotective effects in multiple cardiac disease settings (48).

Collectively, the NLRP3 inflammasome-pyroptosis pathway represents a biologically plausible mechanistic hypothesis for ATO-induced cardiac inflammation and injury, based on conserved oxidative stress-inflammatory cascades and supportive findings from non-cardiac ATO models. However, its definitive causal role and quantitative contribution in ATO-induced cardiotoxicity have not been directly verified via targeted gene knockout or specific inhibitor studies in cardiac models, and therefore remain to be confirmed by further dedicated investigations.

### 4.2 NF-κB pathway-mediated pro-inflammatory cascade

Nuclear factor kappa-B (NF-κB) is the core transcription factor governing inflammatory responses. Under resting conditions, it forms an inactive complex with the inhibitory protein IκB and is sequestered in the cytoplasm. Both oxidative stress and pro-inflammatory signals can activate IκB kinase (IKK). Activated IKK mediates IκB phosphorylation, which targets IκB for degradation via the ubiquitin-proteasome pathway. The released NF-κB subsequently translocates into the nucleus and binds to κB sites on its target genes. This binding event initiates the transcription of a broad range of pro-inflammatory cytokines, adhesion molecules and chemokines (49–51).

In ATO-treated myocardial tissues, the NF-κB pathway is markedly activated, accompanied by upregulation of downstream pro-inflammatory mediators including TNF-α, IL-1β, IL-6 and ICAM-1 (14, 52). These cytokines act on cardiomyocytes through autocrine and paracrine pathways, further inducing apoptosis and impairing contractile function. Concurrently, upregulated adhesion molecules promote neutrophil and monocyte infiltration into myocardial tissue, amplifying the inflammatory cascade. Multiple interventional studies have confirmed that NF-κB inhibition attenuates ATO-induced myocardial inflammation and injury: agents including Shengxian Decoction, baicalin and astragaloside IV exert cardioprotective effects by suppressing NF-κB nuclear translocation and downregulating pro-inflammatory cytokines (14, 53, 54). The NF-κB pathway also upregulates inflammation-associated enzymes such as inducible nitric oxide synthase (iNOS) and cyclooxygenase-2 (COX-2), driving excessive nitric oxide (NO) and prostaglandin production that further exacerbates local tissue damage (55, 56).

## 5 Proposed integrative mechanistic framework: the oxidative stress-inflammation positive feedback loop

Accumulating preclinical evidence from cellular and rodent models indicates that oxidative stress and inflammation do not operate as two fully independent parallel pathways in ATO-induced cardiotoxicity. Instead, multi-layered molecular interactions support a tightly coupled positive-feedback loop that may contribute to cascade-amplified cardiac injury. Oxidative stress may act as an upstream trigger for inflammatory activation, and sustained inflammation may in turn exacerbate oxidative damage through diverse downstream pathways. The two processes may mutually reinforce each other, and this interplay is associated with progression from reversible electrophysiological abnormalities toward irreversible structural remodeling. Of note, most supportive evidence are correlative, and definitive causal proof requires dedicated genetic or target-specific pharmacological interventional studies in ATO-challenged cardiac models. Partial components of this network—such as NF-κB-driven proinflammatory cytokine release—have been verified via pharmacological inhibition in ATO-exposed myocardial tissues (33, 50, 54).

Notably, supportive evidence for this loop presents distinct levels of validation. [Direct cardiac evidence] ATO-exposed cardiomyocytes and myocardial tissues consistently show that ATO induces excessive ROS, suppresses Nrf2-mediated antioxidant defense, and activates NF-κB-driven pro-inflammatory cytokine release (TNF-α, IL-1β, IL-6) (33, 40, 57). [Extrapolated evidence] By contrast, NLRP3 inflammasome involvement in this loop is largely extrapolated from ATO neurotoxicity and other inflammatory cardiovascular models (47, 58), with direct ATO-cardiac validation still lacking. Accordingly, the positive feedback loop represents a potential mechanistic contributor to ATO cardiotoxicity that is consistent with available correlative evidence, but its definitive causal role and quantitative contribution to disease progression await further confirmation via interventional studies using loop-specific targeting agents.

### 5.1 Oxidative stress-driven inflammatory activation

The pro-inflammatory driving effect of oxidative stress manifests across multiple stages of inflammatory initiation and activation. During the inflammatory priming phase, ROS mediates oxidative modification of IκB and IKK. This modification promotes IκB degradation and NF-κB nuclear translocation, and upregulates the gene expression of inflammasome components and pro-inflammatory cytokines. These molecular events complete the priming step of the NLRP3 inflammasome and prepare pro-inflammatory genes for transcription, as a general inflammatory mechanism demonstrated in non-cardiac models (59). Meanwhile, ROS also activates kinases of the mitogen-activated protein kinase (MAPK) family, including JNK and p38. These activated kinases synergistically promote pro-inflammatory gene expression via transcription factors such as AP-1, and further enhance the transcriptional magnitude of inflammatory responses; this MAPK/AP-1 axis has been characterized in vascular smooth muscle and other non-cardiac cell systems (60), and its direct operation in ATO-exposed cardiomyocytes requires further cardiac-specific validation.

During the inflammatory activation phase, ROS acts as one of the most well-characterized activators of the NLRP3 inflammasome. Both mitochondria-derived ROS and NADPH oxidase-derived ROS activate NLRP3 through both direct molecular interactions and indirect downstream signaling. They trigger inflammasome assembly and caspase-1 activation, and serve as a critical bridge linking oxidative stress and inflammasome activation (61). In addition, multiple oxidative stress-induced events function as potent activation signals for the NLRP3 inflammasome. These events include lysosomal membrane damage, mitochondrial DNA release and intracellular potassium efflux, which collectively drive inflammasome activation (62). Taken together, oxidative stress provides both priming signals and activation signals for inflammatory responses. It constitutes an essential prerequisite for the initiation of the entire inflammatory cascade.

### 5.2 Inflammation-exacerbated oxidative damage

Once initiated, inflammatory responses reciprocally aggravate oxidative stress through diverse pathways, further amplifying the severity of oxidative damage. First, pro-inflammatory cytokines suppress the function of the endogenous antioxidant system in cardiomyocytes via receptor-mediated signaling pathways. For instance, TNF-α and IL-1β are associated with downregulated Nrf2 transcriptional activity; in ATO-induced rat myocardial injury, reduction of these cytokines by cardioprotective intervention is accompanied by restored Nrf2 activity and antioxidant enzyme expression (14). This cytokine-mediated suppression of Nrf2 may reduce the expression and activity of antioxidant molecules including SOD, CAT and GSH, impair intrinsic antioxidant defense, and facilitate ROS accumulation. In addition, inflammatory cytokines may disrupt mitochondrial respiratory chain function and promote mitochondrial ROS production, as described in general cardiovascular disease models (63). They may also inhibit mitophagy and result in accumulation of damaged mitochondria, further exacerbating oxidative stress; however, these cytokine-mitochondria interactions have not been directly demonstrated in ATO-exposed cardiac tissue and remain an inferred component of the proposed feedback loop. This amplifying effect of inflammation on oxidative stress may allow sustained oxidative damage even after the initial oxidative stimulus subsides, and is consistent with the progression of chronic myocardial injury, where the proposed feedback loop may become fully established.

### 5.3 Spatiotemporal heterogeneity of the proposed feedback loop

In the proposed phase-specific model described above, the oxidative stress–inflammation positive feedback loop exhibits distinct activation status and functional relevance across the acute and chronic phases of ATO-induced cardiotoxicity. This spatiotemporal framing remains a working hypothesis rather than an established temporal sequence.

In the acute phase, characterized by reversible electrophysiological abnormalities and QTc prolongation, the predominant pathogenic mechanism is direct ATO-mediated ion channel dysfunction, including impaired hERG and Kir2.1 trafficking and intracellular calcium overload (28–31). Oxidative stress may be present as an early event, but the full reciprocal feedback loop—whereby sustained inflammation reciprocally amplifies oxidative damage—appears incompletely established at this stage. The inflammatory response in the acute phase is relatively mild and may function primarily as a downstream consequence of oxidative stress rather than a self-sustaining amplification circuit. Consistent with this, standalone antioxidant strategies show favorable efficacy against acute ATO-induced electrophysiological injury (64), whereas anti-inflammatory monotherapy delivers limited protection against acute high-dose oxidative injury.

In the chronic phase, characterized by persistent mitochondrial dysfunction, progressive cardiomyocyte loss, interstitial fibrosis and structural remodeling, the proposed feedback loop may become fully established and self-sustaining. Persistent ROS production continuously drives inflammatory activation via NF-κB and potentially NLRP3 pathways, while accumulated pro-inflammatory cytokines in turn suppress Nrf2-mediated antioxidant defense and disrupt mitochondrial function, maintaining a sustained high level of both oxidative stress and inflammation (14, 33, 63). This self-amplifying loop may contribute to the irreversibility of chronic structural damage. Consistent with this phase-specific model, dual-target interventions that simultaneously block both arms of the loop are mechanistically justified for chronic structural injury, and several such agents—including Shengxian Decoction, the bisoprolol–trimetazidine combination, and baicalin—have demonstrated protective efficacy in ATO cardiotoxicity models (14, 40, 53). However, direct head-to-head comparisons of dual-target versus single-target strategies remain limited, and definitive evidence of superior efficacy requires further controlled comparative studies.

It should be emphasized that this spatiotemporal model is based on correlative preclinical evidence and remains a proposed framework. Direct longitudinal studies tracking the dynamic activation of both oxidative and inflammatory pathways from acute to chronic phases in the same ATO-exposed cardiac model are needed to definitively validate the phase-specific establishment of the feedback loop.

## 6 Targeted cardioprotective strategies based on the oxidative stress-inflammation axis

Diverse cardioprotective strategies have been developed to target distinct nodes of the oxidative stress-inflammation positive feedback loop. These include standalone antioxidant strategies, standalone anti-inflammatory strategies, and dual intervention strategies that simultaneously target both pathological processes. These strategies have demonstrated varying degrees of protective efficacy in both cellular and animal models. They provide a broad panel of candidate regimens to support subsequent clinical translation. The results are shown in **Table 1**.

**Table 1:** Summary of studies on the cardiotoxicity protection of arsenic trioxide targeting the oxidative stress inflammation axis.

Drug	Targeted strategy	Model	Evidence level	Signaling pathway	Key molecular targets	Main mechanism of action	Reference
Resveratrol	Targeted oxidative stress, calcium overload and apoptosis	H9c2 cardiomyocytes; mouse cardiomyopathy model	*In vitro* + *In vivo*	Oxidative stress-calcium overload-apoptosis axis	ROS, intracellular Ca²^+^, caspase-3, SOD, CAT, GSH-Px	Scavenges reactive oxygen species, restores endogenous antioxidant enzyme activities, inhibits intracellular calcium mobilization and cardiomyocyte apoptosis, alleviates QT interval prolongation and myocardial structural damage	(65)
Ellagic acid	Targeted oxidative stress	Wistar rat cardiotoxicity model	*In vivo*	Antioxidant defense pathway	GPx, MDA, QTc, cTnI, CK-MB	Exerts antioxidant effects, reduces lipid peroxidation level, restores antioxidant enzyme activities, alleviates QTc prolongation and myocardial enzyme release	(66)
Flaxseed oil	Targeted oxidative stress and arsenic accumulation	Wistar rat cardiotoxicity model	*In vivo*	Antioxidant defense pathway	LPO, GSH, SOD, CAT, cardiac arsenic burden	Reduces arsenic deposition in heart tissue, alleviates lipid peroxidation, enhances antioxidant enzyme activities, improves myocardial structural pathological damage	(67)
Protocatechuic acid	Targeted oxidative stress and inflammation	H9c2 cardiomyocytes	*In vitro*	TLR4 signaling pathway	ROS, FRAP, TLR4, hydroperoxides	Enhances total antioxidant capacity, reduces oxidative damage, downregulates TLR4 gene expression, inhibits inflammatory response in cardiomyocytes	(68)
Metallothionein	Targeted oxidative stress, apoptosis and fibrosis	Cardiac-specific metallothionein transgenic mice	*In vivo*	Death receptor pathway; ER stress pathway; NF-κB inflammatory pathway	ROS, NF-κB, inflammatory cytokines, adhesion molecules, chemokines, caspase family	Scavenges oxygen free radicals to reduce oxidative stress injury, inhibits death receptor and endoplasmic reticulum stress apoptotic pathways, suppresses NF-κB-mediated inflammatory response and myocardial fibrosis	(69)
Salvianolic acid A (Sal A)	Targeted calcium homeostasis and endoplasmic reticulum stress	BALB/c mice; adult rat ventricular myocytes	*In vitro* + *In vivo*	SERCA-mediated calcium homeostasis/ER stress pathway	SERCA, [Ca²^+^]ER, ER stress-related proteins, Ca²^+^ overload	Increases SERCA activity and protein expression, restores intracellular calcium homeostasis, downregulates endoplasmic reticulum stress-mediated cardiomyocyte apoptosis	(32)
Piperine	Targeted oxidative stress and inflammation	Wistar rat cardiotoxicity model	*In vivo*	Oxidative stress-inflammatory axis	CK-MB, LDH, TBARS, SOD, CAT, IL-6	Reduces release of cardiac injury biomarkers, alleviates oxidative damage and inflammatory response, shortens ATO-induced prolonged QT/QTc intervals	(70)
6-Gingerol	Targeted oxidative stress, inflammation and mitochondrial biogenesis	Kunming mouse cardiotoxicity model	*In vivo*	AMPK/SIRT1/PGC-1α signaling pathway	AMPK, SIRT1, PGC-1α, ROS, inflammatory cytokines, caspase-3	Activates AMPK/SIRT1/PGC-1α signaling axis, alleviates oxidative stress, inflammatory response and mitochondrial damage, inhibits cardiomyocyte apoptosis	(71)
Hesperidin	Targeted oxidative stress, inflammation and apoptosis	Mouse cardiotoxicity model	*In vivo*	p62-Keap1-Nrf2 signaling pathway	Nrf2, Keap1, p62, ROS, TNF-α, IL-6, Bax/Bcl-2, caspase-3	Activates p62-Keap1-Nrf2 antioxidant pathway, enhances endogenous antioxidant defense, inhibits inflammatory response and mitochondria-dependent apoptosis	(72)
Aloin	Targeted oxidative stress and inflammation	Mouse cardiotoxicity model	*In vivo*	Antioxidant defense pathway	ROS, SOD, CAT, inflammatory cytokines, QT interval	Dose-dependently alleviates ATO-induced oxidative stress, reduces levels of cardiac inflammatory cytokines, improves ECG abnormalities such as ST-segment elevation and QT prolongation	(73)
Sorbus pohuashanensis flavonoids	Targeted oxidative stress and apoptosis	BALB/c mice; H9c2 cells	*In vitro* + *In vivo*	Akt/Nrf2/HO-1 signaling pathway	p-Akt, Nrf2, HO-1, SOD, CAT, GSH-Px, ROS	Upregulates phosphorylated Akt, activates Nrf2/HO-1 antioxidant signaling, enhances antioxidant enzyme activities, inhibits ATO-induced cardiomyocyte apoptosis	(74)
Sal A	Targeted mitochondrial oxidative injury	BALB/c mice; H9c2 cardiomyocytes/tumor cell lines	Mixed — cardiac + non-cardiac (BALB/c mice; H9c2 cardiomyocytes + tumor cell lines)	Mitochondrial protection pathway	mPTP, mitochondrial ROS, ATP, mitochondrial biogenesis-related molecules	Inhibits abnormal opening of mitochondrial permeability transition pore, reduces mitochondrial ROS overproduction, improves ATP generation and mitochondrial biogenesis	(75)
Grape seed and skin extract	Targeted oxidative stress and trace element imbalance	Wistar rat cardiotoxicity model	*In vivo*	Antioxidant defense pathway	LPO, protein carbonylation, SOD, CAT, calcium homeostasis, trace elements	Alleviates lipid peroxidation and protein carbonylation, restores antioxidant enzyme activities, improves calcium and myocardial trace element homeostasis	(76)
Phloretin	Targeted calcium homeostasis and inflammation	H9c2 cardiomyoblasts	*In vitro*	Ca²^+^/Calcineurin/NFATc pathway; MAPK pathway	Intracellular Ca²^+^, calcineurin, NFATc, IL-2, IL-6, TNF-α, MAPK	Inhibits ATO-induced calcium overload and calcineurin/NFATc activation, reduces release of inflammatory cytokines, modulates MAPK signaling pathway	(77)
2-Aminoethoxydiphenyl borate	Targeted oxidative stress and myocardial injury	Sprague-Dawley rat cardiotoxicity model (*in vivo*)	*In vivo*	Antioxidant defense pathway	LDH, CK, cTnI, MDA, SOD, CAT	Alleviates QT interval prolongation and ST-segment abnormalities, reduces release of cardiac injury biomarkers, enhances myocardial antioxidant defense capacity	(78)
Omega-3 fatty acid	Targeted oxidative stress and arsenic accumulation	Wistar rats; H9c2 cardiomyoblasts	*In vitro* + *In vivo*	Antioxidant defense pathway	ROS, calcium homeostasis, mitochondrial membrane potential, cardiac arsenic burden	Reduces arsenic accumulation in cardiac tissue, alleviates oxidative stress, improves intracellular calcium homeostasis and mitochondrial function	(79)
Boerhavia diffusa ethanolic extract	Targeted oxidative stress and organelle damage	H9c2 cardiomyocytes (*in vitro*)	*In vitro*	Oxidative stress-organelle injury axis	ROS, intracellular Ca²^+^, mitochondrial membrane potential, LDH	Scavenges reactive oxygen species, alleviates calcium overload, protects mitochondria, endoplasmic reticulum, lysosomes and other organelles from damage	(80)
Eugenol	Targeted oxidative stress and electrolyte imbalance	Wistar rat cardiotoxicity model (*in vivo*)	*In vivo*	Antioxidant defense/ion homeostasis pathway	LDH, CK-MB, GSH, GST, GPx, Na^+^/K^+^-ATPase, Ca²^+^-ATPase	Enhances myocardial antioxidant system, maintains ion pump activities and electrolyte homeostasis, corrects ATO-induced QT interval prolongation	(81)
Zinc (Zn²^+^)	Targeted oxidative stress, apoptosis and autophagy	Common carp cardiotoxicity model (*in vivo*)	*In vivo*	PI3K/Akt/mTOR pathway; MAPK pathway	ROS, SOD, CAT, MDA, Bax/Bcl-2, caspase family, LC3, p62	Activates antioxidant system via MAPK pathway, inhibits apoptosis and excessive autophagy via PI3K/Akt/mTOR pathway, alleviates myocardial injury	(82)
Salvianolic acid B (Sal B)	Targeted oxidative stress and apoptosis	BALB/c mice; HepG2/HeLa cells	Mixed — cardiac + non-cardiac (BALB/c mice cardiac model; HepG2/HeLa cells are non-cardiac tumor lines)	PI3K/Akt signaling pathway	p-Akt, Bcl-2, caspase-3	Activates PI3K/Akt survival pathway, upregulates anti-apoptotic protein Bcl-2, inhibits cardiomyocyte apoptosis and oxidative damage	(83)
Tannic acid	Targeted oxidative stress, inflammation and mitochondrial apoptosis	Rat cardiotoxicity model	*In vivo*	Mitochondrial apoptosis pathway; NF-κB inflammatory pathway	ROS, MDA, NF-κB p65, Bax/Bcl-2, cytochrome c, caspase-3	Alleviates oxidative stress and inflammatory response, inhibits mitochondria-mediated apoptotic pathway, reduces myocardial pathological damage	(84)
Resveratrol	Targeted oxidative stress and arsenic efflux	Rat cardiotoxicity model	*In vivo*	Nrf2/HO-1 signaling pathway	Nrf2, HO-1, ROS, intracellular calcium, arsenic accumulation	Activates Nrf2/HO-1 antioxidant pathway, maintains cellular redox homeostasis, promotes arsenic efflux, alleviates cardiac dysfunction	(1)
Shengxian Decoction	Targeted oxidative stress, inflammation and mitochondrial biogenesis	Sprague-Dawley rat cardiotoxicity model	*In vivo*	AMPK/SIRT1/PGC-1α signaling pathway; NF-κB pathway	AMPKα2, SIRT1, PGC-1α, NF-κB, ROS, IL-1β, IL-6, TNF-α	Activates AMPK/SIRT1/PGC-1α signaling axis, inhibits NF-κB inflammatory pathway, alleviates oxidative stress, inflammatory response and cardiomyocyte apoptosis	(14)
Pentoxifylline	Targeted oxidative stress	Mouse cardiotoxicity model	*In vivo*	Antioxidant defense pathway	LPO, TAC, TTGs, CAT, SOD, GPx, LDH, CPK, cTnI	Reverses ATO-induced oxidative stress, restores total antioxidant capacity and antioxidant enzyme activities, reduces myocardial histopathological damage	(85)
Bisoprolol + Trimetazidine	Targeted oxidative stress, inflammation and apoptosis	Wistar rat acute myocardial injury model	*In vivo*	PI3K/GSK-3β/Nrf2/HO-1 pathway; NF-κB/iNOS pathway	Nrf2, HO-1, PI3K, NF-κB p65, MDA, inflammatory cytokines, caspase-3	Activates Nrf2 antioxidant pathway, inhibits NF-κB inflammatory signaling, reduces cardiomyocyte apoptosis, synergistically alleviates acute myocardial injury	(40)
L-ascorbic acid + α-tocopherol	Targeted oxidative stress and apoptosis	H9c2 cardiomyocytes (*in vitro*)	*In vitro*	Nrf2/Bcl-2 signaling pathway	Nrf2, Bcl-2, ROS, mitochondrial membrane potential, intracellular calcium	Activates Nrf2 antioxidant signaling and anti-apoptotic Bcl-2 pathway, restores mitochondrial function, inhibits calcium overload and cardiomyocyte apoptosis	(15)
Matrine/Oxymatrine	Targeted hERG channel transcription and trafficking	HEK293-hERG cells; guinea pig ventricular myocytes	*In vitro* — cardiac electrophysiology (HEK293-hERG cells; guinea pig ventricular myocytes)	Sp1-mediated hERG transcription pathway	Sp1, hERG channel, action potential duration	Upregulates transcription factor Sp1, increases hERG channel expression and membrane trafficking, shortens ATO-prolonged action potential duration	(28)
Phloretin	Targeted mitochondrial dysfunction	H9c2 cardiomyoblasts (*in vitro*)	*In vitro*	Mitochondrial ETC/Nrf2 pathway	ETC complexes, mitochondrial membrane potential, mPTP, Nrf2, caspase-3	Protects mitochondrial electron transport chain function, maintains mitochondrial membrane potential, inhibits mPTP opening and cardiomyocyte apoptosis	(36)
Metallothionein	Targeted oxidative stress and MAPK activation	H9c2 cells; MT-transgenic mice	*In vitro* + *In vivo*	MAPK signaling pathway	ERK1/2, JNK, p38, caspase-3, Bax/Bcl-2, CHOP	Inhibits activation of MAPK subfamily members, blocks oxidative stress-mediated apoptotic signaling, reduces ATO-induced cardiomyocyte death	(25)
Sal B	Targeted calcium homeostasis and endoplasmic reticulum stress	BALB/c mice; adult rat ventricular cardiomyocytes	*In vitro* + *In vivo*	SERCA-mediated calcium handling/ER stress pathway	SERCA, Ca²^+^ handling proteins, ERS proteins	Increases SERCA activity, regulates calcium handling protein expression, inhibits endoplasmic reticulum stress, maintains intracellular calcium homeostasis	(2)
Berberine nanoparticles	Targeted oxidative stress	Wistar rat cardiotoxicity model (*in vivo*)	*In vivo*	Antioxidant defense pathway	Cardiac enzymes, MDA, SOD, CAT	Balances myocardial oxidative stress biomarkers, reduces release of cardiac injury enzymes, improves ATO-induced myocardial structural damage	(22)
Polyphenol-rich apple peel extract	Targeted oxidative stress	H9c2 cardiomyocytes	*In vitro*	Antioxidant defense pathway	SOD, CAT, GSH, GPx, ROS, calcium overload, caspase-3	Scavenges reactive oxygen species, restores intracellular antioxidant enzyme system, alleviates calcium overload and cardiomyocyte apoptosis	(86)
Baicalin	Targeted oxidative stress, inflammation and apoptosis	Kunming mouse cardiotoxicity model (*in vivo*)	*In vivo*	TLR4/NF-κB signaling pathway	TLR4, NF-κB, TNF-α, IL-6, Bax/Bcl-2, caspase-3, ROS	Inhibits TLR4/NF-κB inflammatory pathway, alleviates oxidative stress and inflammatory response, reduces ATO-induced cardiomyocyte apoptosis	(53)
Astragaloside IV	Targeted oxidative stress, inflammation and apoptosis	Rat cardiotoxicity model (*in vivo*)	*In vivo*	p38 MAPK/NF-κB signaling pathway	p38 MAPK, NF-κB, MDA, SOD, GSH-Px, IL-1β, TNF-α, Bax/Bcl-2	Inhibits p38 MAPK/NF-κB signaling axis, alleviates oxidative stress–inflammation positive feedback loop, reduces cardiomyocyte apoptosis	(54)
Sal A	Targeted oxidative stress and apoptosis	H9c2 cardiac cells (*in vitro*)	*In vitro*	MAPK signaling pathway	JNK, ERK, p38, Bcl-2, Bax, caspase-3, ROS, ΔΨm	Inhibits MAPK pathway activation, suppresses mitochondrial membrane depolarization, regulates expression of apoptosis-related proteins	(87)
α-Lipoic acid	Targeted arsenic chelation and potassium channel protection	Anesthetized guinea pigs; isolated guinea pig cardiomyocytes	*In vivo* — cardiac electrophysiology (anesthetized guinea pigs; isolated cardiomyocytes)	IKs potassium channel regulation/arsenic chelation	IKs current, QTc interval	Forms complex with arsenic via chelating effect, restores IKs potassium current, rapidly alleviates ATO-induced QTc interval prolongation	(64)
Resveratrol	Targeted hERG channel trafficking deficiency and ER stress	HEK293-hERG cells; guinea pig ventricular myocytes (*in vitro*)	*In vitro*	hERG chaperone complex/ER stress pathway	Hsp70/Hsp90, calnexin, calreticulin, ATF6, hERG	Restores hERG-Hsp70/Hsp90 chaperone complex function, relieves endoplasmic reticulum stress, rescues hERG channel trafficking deficiency	(16)
Sal B	Targeted oxidative stress and apoptosis	H9c2 cardiomyocytes (*in vitro*)	*In vitro*	PI3K/Akt signaling pathway	PI3K, Akt, Bcl-2, Bcl-xl, Bax, caspase-3, ROS	Activates PI3K/Akt survival pathway, upregulates anti-apoptotic proteins, inhibits oxidative stress-induced cardiomyocyte apoptosis	(88)

CHOP, C/EBP-homologous protein; SOD, Superoxide Dismutase; CAT, Catalase; GSH, Glutathione; GPx, Glutathione Peroxidase; Sal A, Salvianolic acid A; Sal B, Salvianolic acid B; LPO, Lipid PerOxide; TAC, total antioxidant capacity; TTGs, total thiol groups; LDH, Lactate Dehydrogenase; CPK, Creatine phosphokinase; cTnI, troponin-I; MDA, Malonaldehyde; ATF6, Activating transcription factor 6; hERG, human ether-a-go-go-related gene. *In vitro* — cardiac: experiments in cardiomyocytes, cardiac cell lines (H9c2, HL-1), or isolated cardiac tissue. *In vivo* — cardiac: rodent or other animal ATO cardiotoxicity model with cardiac readouts.

*In vitro* — non-cardiac: experiments in non-cardiac cell lines (e.g., HepG2, HeLa, RAW 264.7, leukemia cells); mechanistically relevant but extrapolated to the heart. Mixed: combines cardiac and non-cardiac evidence; non-cardiac components should be interpreted as supportive rather than definitive. Clinical: human data in ATO-treated patients. No entry in this table currently reaches clinical evidence level; all listed studies are preclinical.

### 6.1 Standalone antioxidant strategies

Standalone antioxidant strategies primarily exert cardioprotective effects through multiple approaches, including supplementing exogenous antioxidants, activating endogenous antioxidant pathways, and targeting mitochondrial oxidative damage. These interventions directly scavenge ROS and restore redox homeostasis, thereby blocking the initiating step of the cardiotoxicity cascade.

Nrf2 pathway agonists represent the most extensively investigated class of antioxidant protective agents. These agents interfere with Keap1-dependent Nrf2 degradation to stabilize Nrf2 protein. Upon nuclear translocation, Nrf2 binds to ARE sequences and transactivates a panel of downstream antioxidant and phase-II detoxifying effector proteins including HO-1, NQO1, and enzymes supporting GSH biosynthesis, thereby exerting broad-spectrum antioxidant protective effects. Resveratrol is a representative agent of this class. Accumulating studies have verified that resveratrol significantly reverses ATO-induced downregulation of Nrf2 and HO-1 expression, restores myocardial antioxidant enzyme activity, reduces ROS production and lipid peroxidation, and simultaneously ameliorates myocardial pathological damage and cardiac dysfunction (1). Similar natural polyphenols, including phloretin, apple peel extract and eugenol, also exert protective effects against ATO-induced cardiotoxicity via activation of the Nrf2 pathway (36, 86, 89).

Mitochondria-targeted antioxidants act directly on mitochondria, the primary source of oxidative stress. They reduce ROS production by preserving mitochondrial structure and function. α-Lipoic acid is a representative agent in this category. It significantly ameliorates ATO-induced QT interval prolongation by chelating ATO and restoring IKs current, and exerts dual preventive and therapeutic effects (64).

### 6.2 Standalone anti-inflammatory strategies

Standalone anti-inflammatory strategies primarily exert cardioprotective effects through multiple approaches, including inhibiting inflammasomes, blocking the NF-κB pathway, and antagonizing pro-inflammatory cytokines. These interventions alleviate inflammation-mediated myocardial injury and primarily target the amplification step of the cardiotoxicity cascade.

NLRP3 inflammasome-specific inhibitors such as MCC950 selectively block the ATPase activity of NLRP3, thereby inhibiting inflammasome assembly and the maturation and release of IL-1β and IL-18. MCC950 has demonstrated significant anti-inflammatory and cardioprotective effects in other cardiovascular disease models (48). Although direct investigations of its efficacy against ATO-induced cardiotoxicity remain limited, this class of inhibitors holds potential therapeutic value based on the mediating role of inflammasomes in ATO cardiotoxicity. NF-κB pathway inhibitors represent a more well-established anti-inflammatory strategy. Pentoxifylline suppresses NF-κB activation, reduces pro-inflammatory cytokine release and inflammatory cell infiltration, and significantly mitigates ATO-induced myocardial enzyme elevation and pathological damage (85). In addition, glucocorticoids exert potent anti-inflammatory effects. However, their clinical application is strictly restricted in patients with malignancies due to immunosuppressive side effects and potential tumor-promoting risks.

The core limitation of standalone anti-inflammatory strategies lies in their inability to block the initiating source of oxidative stress. They only alleviate inflammation-mediated secondary damage. These strategies deliver limited protective efficacy against oxidative injury induced by acute high-dose ATO exposure. Meanwhile, long-term suppression of inflammatory responses may impair the organism’s immune defense and tissue repair capacities, leading to adverse outcomes including elevated infection risk and delayed tissue healing.

### 6.3 Dual-target combination and single-agent strategies

Given the proposed positive feedback relationship between oxidative stress and inflammation, dual intervention strategies that simultaneously target both pathological processes carry a strong mechanistic rationale: blocking one arm of a self-amplifying loop may leave the other arm able to reinitiate injury. This rationale, however, is distinct from demonstrated comparative efficacy. To date, several dual-target agents have shown cardioprotective activity in ATO cardiotoxicity models when tested against vehicle controls. Crucially, no study has yet directly compared a dual-target strategy with a matched single-target arm (e.g., antioxidant alone vs. anti-inflammatory alone vs. combined) in the same ATO cardiac model, with sample size powered to detect superiority. The perception that dual-target approaches outperform single-target ones therefore remains an inference from mechanistic logic, not an experimentally established comparative finding. Dual intervention can be achieved either through the combination of two separate therapeutic agents, or via a single agent with intrinsic antioxidant and anti-inflammatory activities.

Natural products represent a critical source of dual-target protective agents. Numerous plant-derived polyphenols and flavonoids possess dual activities of free radical scavenging and inflammatory pathway suppression. Resveratrol not only exerts antioxidant effects by activating the Nrf2 pathway, but also reduces pro-inflammatory cytokine release via inhibition of the NF-κB pathway. It simultaneously ameliorates endoplasmic reticulum stress and ion channel dysfunction, thus blocking the cardiotoxicity cascade at multiple regulatory nodes (1, 16). Salvianolic acid A and salvianolic acid B are the primary active constituents of Salvia miltiorrhiza. They exert multi-target cardioprotective effects through multiple mechanisms. These include inhibiting cardiomyocyte apoptosis via the phosphatidylinositol 3-kinase/protein kinase B (PI3K/Akt) pathway, regulating calcium homeostasis, alleviating endoplasmic reticulum stress, and suppressing inflammatory activation of the MAPK and NF-κB pathways (2, 87, 88). In addition, traditional Chinese medicine monomers and herbal formulas including baicalin, astragaloside IV and Shengxian Decoction exert protective effects against ATO-induced cardiotoxicity, with *in vitro*/*in vivo* assays showing concurrent antioxidant and anti-inflammatory activities. The word “synergistic” should be read descriptively (i.e., the agent engages both pathways) rather than as evidence that the combination is quantitatively superior to either activity alone; formal isobolographic or dose-matrix synergy analysis against matched single-pathway controls has not been performed in ATO cardiac models (14, 53, 54).

For example, metformin, a well-established first-line anti-diabetic agent, reduces ROS generation by inhibiting mitochondrial respiratory chain complex I. It concurrently suppresses inflammatory responses via activation of the adenosine monophosphate-activated protein kinase (AMPK) pathway. Notably, its reported protective efficacy was observed in non-cardiac hepatic injury models (90); direct protective effects of metformin against ATO-mediated myocardial damage have not yet been verified in cardiac-relevant experimental systems. Therefore, its therapeutic potential for ATO-induced cardiotoxicity merits further dedicated cardiac-focused exploration. The combination regimen of bisoprolol and trimetazidine simultaneously modulates the PI3K/GSK-3β/Nrf2/HO-1 and NF-κB/iNOS pathways and alleviates ATO-induced acute myocardial injury in rats (40). This study compared the combination against ATO-only vehicle, not against bisoprolol alone or trimetazidine alone; therefore, the incremental contribution of adding the second agent cannot be inferred from these data alone. Both agents have established cardiovascular safety profiles.

### 6.4 Critical comparative assessment of cardioprotective strategies

Current cardioprotective regimens against ATO-induced cardiotoxicity vary substantially in evidence strength, applicable injury phase and impact on antitumor efficacy. A critical comparative analysis is provided below to clarify their respective advantages and translational limitations.

If the proposed phase-specific framework is valid, intervention strategies should be stratified according to injury phase: for acute electrophysiological injury, where the full feedback loop appears incompletely established and ion channel dysfunction dominates, standalone antioxidant or ion-channel-targeted strategies may be more appropriate; for chronic structural injury, where the loop is proposed to be more fully engaged, dual-target interventions that block both arms may be preferable. These stratification recommendations remain mechanistically reasoned proposals and have not been validated in prospective phase-stratified clinical trials. For acute electrophysiological injury, where the full feedback loop appears incompletely established and ion channel dysfunction dominates, standalone antioxidant or ion-channel-targeted strategies may be sufficient. For chronic structural remodeling, where the loop may become self-sustaining, dual-target interventions simultaneously blocking both oxidative and inflammatory arms are mechanistically justified and have demonstrated protective efficacy in preclinical models. Direct comparative studies confirming superior efficacy over single-target approaches are warranted. This phase-stratified approach aligns intervention selection with the differential establishment of the proposed feedback loop.

According to the source and robustness of experimental validation, existing protective strategies can be stratified into three evidence levels. Regimens with robust cardiac-specific validation represent the strongest evidence tier. Agents including resveratrol, salvianolic acid A/B, baicalin and crocin have been verified in both H9c2 cardiomyocyte models and rodent *in vivo* cardiotoxicity models. They consistently show reduced ROS levels, suppressed inflammatory responses and attenuated myocardial pathological damage (1, 2, 50, 57, 81, 88, 90). Most of these agents have been tested in multiple independent laboratories with good reproducibility. Regimens with moderate evidence are those validated only in a single experimental system or with limited mechanistic data. For example, piperine, 6-gingerol and hesperidin have only been tested in rodent *in vivo* models without complementary cellular mechanistic validation, or have only explored a single signaling axis (70–72). Regimens with indirect evidence are inferred from non-cardiac models. For instance, metformin has only been confirmed to protect against ATO-induced liver injury via antioxidant and anti-inflammatory mechanisms (90).

Notably, all currently available evidence is limited to cellular and small rodent models. No large-animal validation or clinical trial data are available for any of these regimens, which represents a common translational bottleneck.

### 6.5 Current status and challenges in clinical translation

Current prevention and management of ATO-induced cardiotoxicity remain predominantly based on symptomatic and supportive care. Routine clinical measures include close monitoring of electrocardiograms and myocardial enzyme levels during treatment, correction of electrolyte disturbances, and avoidance of concurrent administration of other QT-prolonging agents. No targeted etiological interventional drugs are available for clinical use at present (7, 16). Multiple protective agents, including resveratrol, salvianolic acids, and vitamins, have exhibited definite cardioprotective effects in preclinical studies. Nevertheless, most relevant research is still limited to cellular and rodent models, and validation from large animal experiments and clinical studies remains insufficient.

The core barrier to clinical translation is whether cardioprotective agents interfere with the anti-tumor efficacy of ATO. An ideal cardioprotective agent should selectively protect normal cardiomyocytes without impairing the tumoricidal effect of ATO, and may even augment its anti-tumor activity. Available studies demonstrate that agents including resveratrol, salvianolic acid B and vitamin C enhance the sensitivity of tumor cells to ATO via distinct mechanisms while exerting cardioprotective effects. These agents achieve the dual benefit of toxicity attenuation and efficacy potentiation (1, 88, 91), which provides a favorable rationale for clinical combination regimens. In addition, optimization of administration timing and dosage represents another critical determinant of successful translation. Prophylactic administration is the primary strategy for acute toxicity, while chronic toxicity requires long-term maintenance intervention. Individualized treatment regimens should be developed according to specific clinical scenarios.

## 7 Translational biomarkers for early risk stratification

Accurate early risk stratification is critical for optimizing ATO treatment regimens and preventing severe cardiotoxic events. Current clinical monitoring relies predominantly on conventional cardiac markers, while mechanism-based candidate biomarkers reflecting oxidative stress and inflammatory activation are emerging as promising complements. To make the evidence status of each biomarker transparent, we organize this section into three tiers of clinical readiness: Tier 1 — Established clinical monitoring parameters: used in routine patient care with guideline-supported cutoffs (ECG/QTc, cardiac injury enzymes). Tier 2 — Candidate circulating biomarkers: measurable in blood or biofluid, with preliminary human or translational data, but lacking validated cutoffs in ATO-treated populations (circulating oxidative stress products, pro-inflammatory cytokines). Tier 3 — Experimental mechanistic readouts: validated in preclinical models or tissue samples, not yet suitable for routine clinical monitoring (myocardial tissue markers, intracellular pathway readouts, non-coding RNAs).

### 7.1 Tier 1 — established clinical monitoring parameters

Electrocardiographic QTc interval prolongation is the most widely used clinical biomarker for acute ATO cardiotoxicity monitoring. Multiple clinical guidelines recommend continuous ECG monitoring during ATO infusion, with defined risk thresholds: QTc > 500 ms or an absolute increase of > 60 ms from baseline indicates significantly elevated risk of malignant arrhythmia such as torsades de pointes (17, 19, 20). However, QTc prolongation is a functional phenotype that appears only after ion channel dysfunction has occurred, and it cannot predict subclinical myocardial injury before electrophysiological abnormalities develop.

### 7.2 Tier 2 — candidate circulating biomarkers

Given the central role of the oxidative stress-inflammation positive feedback loop in ATO cardiotoxicity, molecular markers reflecting the activation status of this axis hold great translational potential as early warning biomarkers.

Oxidative stress-related biomarkers represent the most extensively studied preclinical candidates. Circulating or myocardial malondialdehyde (MDA), a terminal product of lipid peroxidation, is consistently elevated in ATO-exposed animal models and correlates with the degree of oxidative damage (72). Conversely, reduced glutathione (GSH) content and the GSH/GSSG ratio decrease dose-dependently under ATO exposure, reflecting depletion of the endogenous antioxidant reserve (54, 89). Antioxidant enzymes including superoxide dismutase (SOD) and catalase (CAT), as well as downstream effector heme oxygenase-1 (HO-1), also show dynamic changes that mirror the intensity of oxidative stress (74). Although these are measurable in circulation, their sensitivity, specificity, and clinically actionable thresholds for ATO cardiotoxicity have not been prospectively validated.

Inflammatory biomarkers constitute another important category of early candidates. Pro-inflammatory cytokines including TNF-α, IL-1β and IL-6 are significantly upregulated in myocardial tissue and circulation during ATO-induced cardiac injury, and their elevation precedes the increase of conventional myocardial enzymes in preclinical models (92, 93). These are Tier 2 candidates: they are measurable in blood, but they are non-specific (elevated in sepsis, malignancy, infection) and no ATO-specific cutoff has been established.

In addition, cell death-related molecular markers and non-coding RNAs are emerging as novel candidate biomarkers. Apoptosis-related molecules such as cleaved caspase-3 and the Bax/Bcl-2 ratio sensitively reflect the degree of cardiomyocyte programmed death (14, 88). Long non-coding RNAs such as MALAT1 have been found to regulate hERG channel expression in ATO cardiotoxicity, showing potential as functional biomarkers for electrophysiological injury (30). However, these markers remain at the preclinical exploratory stage.

### 7.3 Tier 3 — experimental mechanistic readouts

Tier 3 markers carry valuable mechanistic information but are not yet suitable for routine clinical monitoring. Their use requires tissue biopsy or complex assays, or they remain at an exploratory stage. Myocardial tissue readouts include cleaved caspase-3, the Bax to Bcl-2 ratio, myocardial MDA and myocardial GSH. These are measured in animal myocardium or in endomyocardial biopsy samples under research conditions (14, 72, 88). Intracellular pathway readouts encompass NF-κB nuclear translocation, NLRP3 inflammasome assembly and phosphorylated Nrf2. These are typically evaluated by Western blot, immunohistochemistry or immunofluorescence in experimental samples. Regarding non-coding RNAs, long non-coding RNA MALAT1 has been linked to hERG regulation in preclinical models of ATO cardiotoxicity (30). Other candidate non-coding RNAs remain in discovery-phase studies. None of these Tier 3 markers has been validated as a clinical decision tool in patients receiving ATO treatment.

### 7.4 Clinical utility and current evidence limitations

From a clinical perspective, the ideal biomarker panel for ATO cardiotoxicity should combine conventional functional markers (QTc interval), structural injury markers (cTnI, CK-MB) and mechanism-based early warning markers (oxidative stress and inflammatory cytokines). This combined strategy can achieve staged risk stratification: oxidative and inflammatory markers for early preclinical warning, QTc monitoring for acute electrophysiological risk assessment, and myocardial enzymes for structural damage confirmation.

Nevertheless, several critical limitations restrict the clinical translation of current biomarkers. First, most mechanism-derived biomarkers (oxidative stress and inflammatory markers) have only been validated in cellular and rodent models, with scarce data from clinical patient cohorts. Their sensitivity and specificity in human ATO-treated populations remain to be confirmed. Second, many of these markers lack cardiac specificity. Systemic oxidative stress and inflammatory responses caused by extra-cardiac conditions may interfere with the judgment of cardiac-specific injury. Third, there are no standardized detection protocols and clinically validated cutoff values for most candidate biomarkers, which hinders their routine clinical application.

In summary, only Tier 1 markers (ECG/QTc, cTnI, CK-MB) are currently actionable in routine ATO clinical care. Tier 2 circulating markers remain research tools requiring prospective validation, and Tier 3 mechanistic readouts are predominantly preclinical. The ideal risk-stratification panel of the future will combine all three tiers, but Tier 2 and Tier 3 elements should not yet be represented as established clinical biomarkers.

## 8 Challenges and future perspectives

### 8.1 Current research bottlenecks

Although accumulating studies have preliminarily delineated the mechanistic role of the oxidative stress-inflammation axis in ATO-induced cardiotoxicity, multiple bottlenecks still impede the implementation of precise clinical cardioprotective strategies. First, notable discrepancies exist between experimental animal models and actual clinical settings. Most preclinical studies are performed in healthy young animals, with drug administration protocols and dosages that differ from standard clinical regimens. These models cannot fully recapitulate the pathophysiological state of patients with cancer, particularly elderly patients and those with underlying cardiovascular comorbidities. This mismatch contributes to the low translational efficiency of results from animal experiments to clinical practice (9, 64).

Second, the dose-response relationship and temporal dynamic characteristics of ATO-induced cardiotoxicity remain poorly elucidated. ATO-induced cardiotoxicity exhibits prominent dose- and time-dependent properties. Nevertheless, the dose thresholds and time windows for distinct injury phenotypes have not been precisely delineated. Furthermore, the dynamic crosstalk between oxidative stress and inflammation has not been fully characterized at the spatiotemporal level. These knowledge gaps hinder the formulation of clinical early warning strategies and stratified intervention regimens.

Third, specific biomarkers for ATO-induced cardiotoxicity remain largely unavailable. Current clinical monitoring of ATO-induced cardiotoxicity predominantly relies on electrocardiograms and myocardial enzyme panels. These markers exhibit limited sensitivity and specificity, as abnormal findings are typically detectable only after definitive myocardial damage has developed. This limitation prevents effective early warning of cardiotoxicity. Specific biomarkers capable of reflecting the activation status of the oxidative stress-inflammation loop, including specific oxidation products, inflammatory cytokines and non-coding RNAs, still require further comprehensive screening and validation.

### 8.2 Future research directions

To address the aforementioned research bottlenecks, future investigations can be advanced along several core directions. First, spatiotemporal dynamic regulatory mechanisms should be comprehensively delineated via multi-omics approaches. The integrated application of transcriptomics, proteomics, metabolomics and spatial omics enables dynamic profiling of the molecular landscape of oxidative stress and inflammation throughout the progression of ATO-induced cardiac injury. This strategy supports the identification of key regulatory nodes across distinct time points and cell subtypes, and comprehensively unravels the dynamic evolution pattern of the positive feedback loop.

Second, myocardium-targeted drug delivery systems should be developed. Technologies including nanocarriers and myocardial targeting peptides enable specific delivery of cardioprotective agents to myocardial tissue. This strategy increases local drug concentration while reducing systemic exposure. It not only enhances cardioprotective efficacy, but also avoids interference with the systemic antitumor activity of ATO. For instance, berberine-loaded nanoparticles have exhibited superior cardioprotective activity compared with free drugs in animal models, confirming the distinct advantages of targeted delivery strategies (22).

Third, high-quality clinical translational research should be advanced. Rigorously designed prospective cohort studies should be conducted to validate the safety and efficacy of candidate cardioprotective agents in clinical populations, and to clarify the optimal dosage, administration timing and treatment duration. Meanwhile, a multicenter registry database for ATO-induced cardiotoxicity should be established to accumulate real-world data, which provides evidence-based rationale for the optimization of cardioprotective regimens.

## 9 Conclusion

ATO-induced cardiotoxicity, proposed to manifest as acute electrophysiological dysfunction and chronic structural remodeling, remains a critical limitation to its broad clinical anti-tumor application. This narrative review proposes the oxidative stress-inflammation positive feedback loop as a possible integrative mechanistic framework that may contribute to ATO cardiotoxicity progression, complementing the conventional view of two independent pathogenic pathways. This framework offers a unifying perspective for interpreting available preclinical evidence, while its definitive causal status remains to be established by dedicated genetic and pharmacological interventional studies.

Within this framework, oxidative stress may act as an upstream initiating event. ATO induces mitochondrial respiratory chain impairment and mitochondrial permeability transition pore opening to generate excess ROS, suppresses the Nrf2/HO-1-mediated endogenous antioxidant system, and may amplify oxidative damage via endoplasmic reticulum stress. ROS may subsequently promote inflammatory activation through two axes: NF-κB-mediated upregulation of NLRP3 and pro-IL-1β primes the NLRP3 inflammasome, and ROS may further trigger inflammasome assembly to activate caspase-1/gasdermin-D-mediated pyroptosis, though direct cardiac validation of this pyroptosis branch remains limited. Reciprocally, pro-inflammatory cytokines such as TNF-α and IL-1β may inhibit Nrf2 transcriptional activity and disrupt mitochondrial function, consistent with a self-amplifying closed-loop circuit. Notably, this proposed loop exhibits spatiotemporal heterogeneity: it appears incompletely established in acute injury dominated by ion channel dysfunction, but may serve as a self-sustaining contributor to chronic myocardial hypertrophy and interstitial fibrosis.

From a translational perspective, dual-target interventions with combined antioxidant and anti-inflammatory activities—represented by natural polyphenols and repurposed agents—have demonstrated cardioprotective efficacy in preclinical models and are mechanistically well-positioned for chronic injury, though direct head-to-head evidence confirming their superiority over single-target strategies remains limited. Several candidates have been reported to preserve ATO’s anti-tumor efficacy. These findings support the heuristic value of the proposed framework for guiding cardioprotective drug development. Future research should focus on cardiac-specific causal validation of the feedback loop, development of standardized biomarker panels, and myocardium-targeted delivery systems, to advance precise clinical cardioprotection for ATO-based chemotherapy.
